# Biofeedback for the Treatment of Stress Urinary Incontinence: A Viable option for Select Patients

**DOI:** 10.5812/numonthly.3382

**Published:** 2012-03-01

**Authors:** Benjamin Dillon, Philippe Zimmern

**Affiliations:** 1 Department of Urology, University of Texas Southwestern, Dallas, Texas, USA

**Keywords:** Urinary Incontinence, Stress, Therapeutics

This is an interesting retrospective study assessing the use of biofeedback for the treatment of stress urinary incontinence (SUI) using ICIQ-SF scores and urodynamics (UDS) parameters as outcome measures (1). The authors concluded that women with SUI improved with biofeedback (BF) techniques. Furthermore, UDS may not be necessary in patients who elect to have BF to manage their SUI, since no UDS changes were noted after BF. We congratulate and commend the authors on their manuscript, as this is a challenging and important topic in our field.

The authors indicated that all of their patients had “genuine stress incontinence.” We feel that this term, although sufficient for this manuscript, should be defined more precisely. “Genuine SUI” can mean many different things to different people, and it is imperative that we understand what “genuine SUI” means. SUI should be defined by both the presence and severity of symptoms (2). The presence of SUI can be identified through a physical exam or UDS while the severity of leakage can be determined by a voiding diary, pad weight test or validated questionnaires. Defining a cure for SUI remains a challenge and is based on the outcome measure(s) selected at the outset of the trial (3). There needs to be an understanding and agreement on a definition of SUI. We feel it is crucial to accurately and subjectively define and quantify SUI prior to any discussion of treatment.

Similarly, when performing UDS testing, we recommend and advocate testing based on ICS standards, which do not appear to have been used in this particular study. There is published level I evidence from the Urinary Incontinence Treatment Network (UITN) (an NIh funded group to research urinary incontinence) on the importance of using a standard protocol when performing UDS. Specifically, the UITN developed a UDS protocol for women with stress predominant SUI that included specific details for annotation, patient positioning, equipment, calibration and data recording (4). Additionally, they published interpretation guidelines to allow central reviewing of the UDS tracings (5, 6). Employing these guidelines would ensure uniformity and accuracy in performing and interpreting UDS for patients with SUI before and after their biofeedback sessions.

We acknowledge the difficulty in asking patients to perform biofeedback exercises when not under the direct supervision of trained physical therapists. however, providing patients with a “workout regimen”, such as how many times per day to perform certain daily exercises and for how long provides a minimum number of exercises for the patient to perform. We have found that advising patients to perform exercises on their own can be confusing and sometimes frustrating for patients. lastly, we agree with the authors on the utility and convenience of using the ICIQ-SF to assess symptoms and quality of life. however, to eliminate recall bias, questionnaires should be administered at the same interval for each patient after completion of their biofeedback treatment.

The results, nonetheless, are encouraging and provide important data to our field. We hope this manuscript motivates other investigators to further expand upon this study or at the very least re-confirm the lack of UDS impact in women treated with BF for bothersome SUI. Truthfully, a multicenter study would be ideal to reproduce these findings and make them more generalizable.
